# The fungal sexual revolution continues: discovery of sexual development in members of the genus *Aspergillus* and its consequences

**DOI:** 10.1186/s40694-020-00107-y

**Published:** 2020-12-24

**Authors:** Valeria Ellena, Michael Sauer, Matthias G. Steiger

**Affiliations:** 1 Austrian Centre of Industrial Biotechnology (ACIB GmbH), Muthgasse 18, Vienna, Austria; 2 Institute of Chemical, Environmental and Bioscience Engineering, TU Wien, Vienna, Austria; 3 Institute of Microbiology and Microbial Biotechnology, BOKU-VIBT, University of Natural Resources and Life Sciences, Muthgasse 18, Vienna, Austria; 4 CD Laboratory for Biotechnology of Glycerol, Muthgasse 18, Vienna, Austria

**Keywords:** Fungal reproduction, *Aspergillus*, *Aspergillus niger*, sexual cycle

## Abstract

Asexuality was considered to be a common feature of a large part of fungi, including those of the genus *Aspergillus*. However, recent advances and the available genomic and genetic engineering technologies allowed to gather more and more indications of a hidden sexuality in fungi previously considered asexual. In parallel, the acquired knowledge of the most suitable conditions for crossings was shown to be crucial to effectively promote sexual reproduction in the laboratory. These discoveries not only have consequences on our knowledge of the biological processes ongoing in nature, questioning if truly asexual fungal species exist, but they also have important implications on other research areas. For instance, the presence of sexuality in certain fungi can have effects on their pathogenicity or on shaping the ecosystem that they normally colonize. For these reasons, further investigations of the sexual potential of *Aspergillus* species, such as the industrially important *A. niger*, will be carried on.

## What is reproduction?

**Fig. 1:**
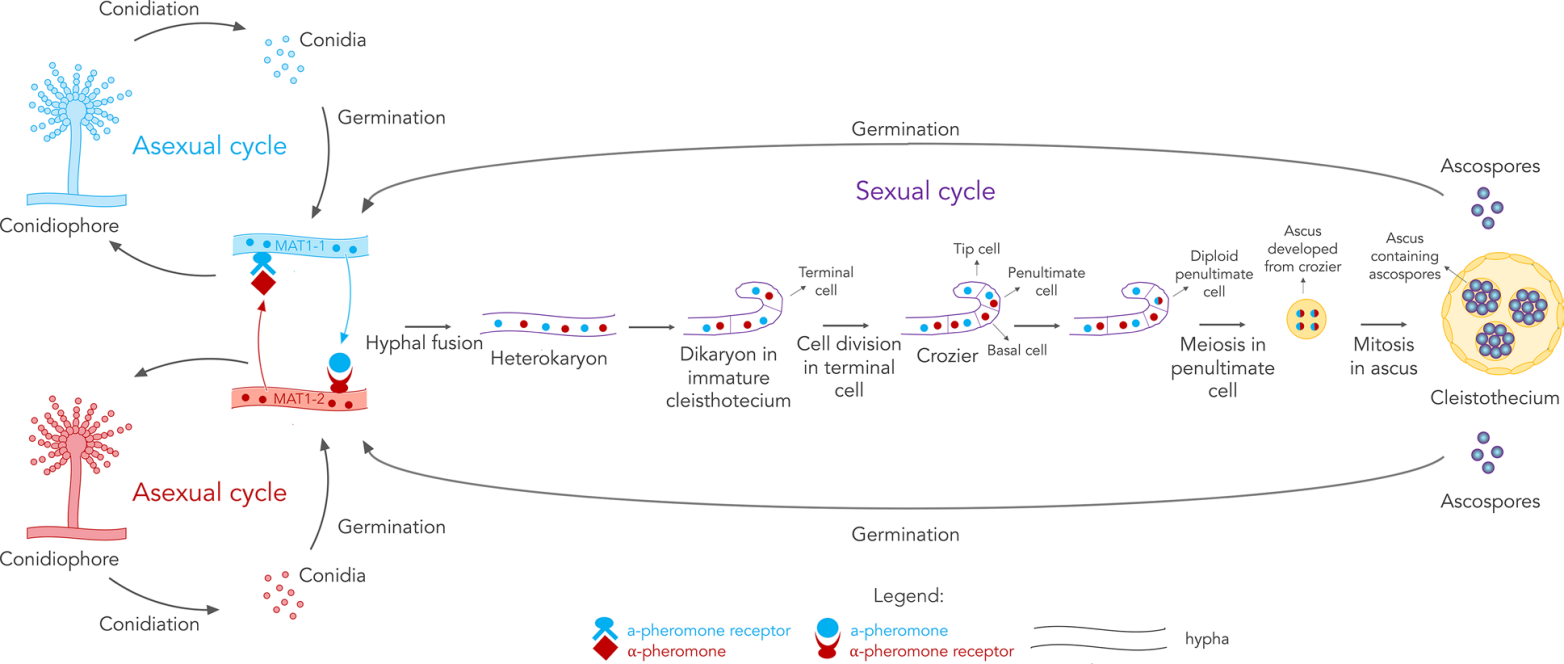
Schematic overview of the asexual and sexual development in the aspergilli and their interconnection [3, 4]. For a more detailed description of the process the reader is invited to visit the poster associated with this publication (Additional file 1)

Reproduction is one of the most fascinating and intriguing processes in nature. Reproduction, is the biological process through which new individuals (offspring) are generated. Not only does it enable a species to be maintained, it is also fundamental for a species to evolve. It is during reproduction that changes by mutations or by reshuffling of genes can be propagated in a species, allowing natural selection and evolution to take place. In nature, and in the fungal world in particular, the modes of reproduction and the frequencies at which these occur are highly diverse [1, 2]. Some organisms, like the *Glomeromycota* fungi, are capable of exclusive clonal reproduction, others, such as mammals, of exclusive sexual reproduction, others, including ciliates and a large group of fungi, alternate these two modes [2]. Within clonal and sexual reproduction, then, specific modes can be observed, depending on the organism [1]. Yeasts, for instance, can reproduce asexually either by budding or fission [1] and sexual reproduction in fungi can occur by self-crossing or outcrossing [2]. An overview of asexual and sexual reproduction in the fungal genus *Aspergillus* is illustrated in Fig. 1.

Both asexual and sexual development provide certain benefits and are associated to certain costs for an organism [1]. The majority of the known eukaryotes go through a sexual phase during their lives and this has led to various debates and discussions about the origin and the evolutionary advantage of such a dispendious mechanism of reproduction [2, 3, 5–8]. Although sexual development is associated with many costs, including the need of finding a partner and the transmission of just half of the parental genes to the progeny [1], it is also known to provide certain advantages to a species. First of all, it is the main source of genetic variation, which occurs mainly through recombination during meiosis but also through the process of crossover [9, 10]. These mechanisms can increase the fitness of an organism and provide adaptation to environmental changes [11]. Secondly, it allows to repair genetic damages by recombination, to mask lethal mutations and to avoid their fixation in the genome [10]. Finally, the formation of highly resistant sexual structures and sexual spores can provide an additional advantage to an organism in the presence of adverse environmental conditions [10]. Despite all these benefits and its widespread presence among eukaryotes, it is quite surprising that for a large group of fungi a sexual cycle has not been observed. In the genus *Aspergillus*, just slightly more than one third of the described species were proven to be sexual [12, 13]. The scope of this primer is to provide an overview of the recent advances and the future perspectives in the discovery and in the study of sexuality in members of the genus *Aspergillus*, with a focus on the important industrial species *Aspergillus niger*.

## Why study sexual reproduction in the aspergilli?

The discovery of sexual cycles in fungi so far considered asexual has major implications. Firstly, it broadens the biological knowledge of the organism and the species, making it possible to perform crossing experiments and classical genetics studies [14]. In addition, the possibility of mating strains in the laboratory is a powerful tool for strain improvement of industrially relevant microorganisms [15]. This process allows the generation of progeny with various combinations of desirable characteristics, without previous knowledge of the genetic basis of the traits of interest. Furthermore, novel strains generated by breeding are considered non genetically modified [16]. Thus, they can be more easily employed as cell factories in the food and feed industry [17]. This is particularly relevant for instance for *A. niger*. Also, it was previously suggested that recombination derived from meiosis and the mating-type might play a role in the increased pathogenicity of certain *Aspergillus* isolates, such as *Aspergillus fumigatus* [18]. Therefore, the possibility of sexuality in natural isolates of *Aspergillus* species is relevant, due to its potential medical implications. Finally, the discovery of a sexual cycle in *A. niger* and other aspergilli might help to better define the concept of species in the genus, since the most commonly accepted and intuitive species concept is based on the ability of two individuals belonging to the same species to breed and produce fertile offspring [19].

## Three advances of research in the last decade

### Advances in the genomic technologies show evidence of sexual potential

Studies and discoveries of the sexual reproduction of certain members of the aspergilli were conducted already in the previous century. For instance, in 1959 the formation of ascospores in *Aspergillus alliaceus* was reported by Fennel and Warcup [20]. Recently, indications of sexuality in fungal species previously considered asexual have been accumulated and complete sexual cycles were found for several of them [15], as shown in Table 1 for a selection of aspergilli.

**Table 1:** Sexual development was discovered in an increasing number of aspergilli important to humankind

Species	Sexual cycle
*A. flavus*	Yes [21]
*A. parasiticus*	Yes [22]
*A. terreus*	Yes [23]
*A. fumigatus*	Yes [24]
*A. nomius*	Yes [25]
*A. tubingensis*	Yes [26]
*A. niger*	No
*A. oryzae*	No
*A. sojae*	No

These discoveries have been facilitated by the recent advances in the genomic technologies. In the last decade, the number of complete fungal genome sequences increased dramatically, aided by the rapid development of the next-generation sequencing (NGS) technologies and the increasing availability of databases and bioinformatics tools for their analysis [27]. This allowed the detection of sex-related genes, particularly the mating-type genes, in presumably asexual species [10], including *A. niger* [28]. The availability of genome sequences of several species also allowed comparative genomics studies so that more than 70 genes proven to be involved in the sexual development of *Aspergillus nidulans*, *A. fumigatus*, *Aspergillus flavus* and *Aspergillus parasiticus* [11] could be identified in other *Aspergillus* species [28–31], providing an excellent basis to study the genetics of sexual development in other aspergilli as well.

Besides the identification of the genes, the availability of genome sequences allows the design of mating-type specific primers, which can be used to rapidly distinguish opposite mating-type strains. Therefore, targeted crossings between opposite mating-type strains can be easily set-up [32]. Next to the detection of sex-related genes, the advances in transcriptomics and the increasing availability of RNA-Seq data also allows the investigation of whether these genes are transcribed and to get an insight into their functionality. For instance, a transcriptome analysis was performed in *Aspergillus cristatus* by comparing gene expression in mating inducive and non-inducive conditions, revealing a great number of MAT target genes [33]. In *A. niger*, not only the mating-type gene MAT1-1, the pheromone precursor gene *ppgA* and the pheromone receptors genes *preA* and *preB* were detected [28, 34], but they were also found to be expressed [31], adding a further indication of a cryptic sexuality for this fungus.

### Advances in the genetic engineering technologies to study sexual reproduction

In parallel with the advances in the genome sequencing and analysis technologies, also the genetic engineering technologies saw a remarkable development in the last years.

Novel cloning strategies, such as the Golden Gate cloning method [35–37], could be successfully applied for the generation of constructs to be used in *A. niger* [38]. CRISPR/Cas9 has also revolutionized genetic engineering in filamentous fungi [39] and can be used to efficiently generate mutants in *A. niger* [38]. Also, the availability of inducible promoter systems greatly facilitates overexpression studies [40]. While all of these technologies can be applied for instance for pathway integration or strain improvement, they can also provide powerful tools to study the sexual potential of the aspergilli. Genetic engineering strategies allowed the confirmation of the functionality of the MAT genes in the sexual development of *A. fumigatus* [41, 42], before a sexual cycle could be described for this species [24]. Isogenic strains differing only in the MAT gene were obtained in *A. oryzae*, making it possible to analyze their expression and their target genes [43]. MAT1-1 and MAT1-2 overexpression strains could be obtained in *A. fumigatus*, showing mating-type dependent expression of pheromone and pheromone precursor genes [44]. The inactivation of the transcription factor *sclB* in *A. niger* was shown to induce the formation of sclerotia [45], which act as sexual structures in species related to *A. niger* [21, 22, 26], and could provide a potential genetic engineering strategy for the generation of strains able to undergo sexual reproduction.

### Importance of media and conditions for the induction of sexual development

The conditions at which the strains are grown are well known to have a crucial impact on fungal growth and development. In the past, environmental conditions were shown to impact not only vegetative growth and asexual development, but also the possibility of certain species to form sclerotia or other reproductive structures [46–48]. Particularly relevant for the induction of a sexual cycle in the aspergilli is the choice of a suitable medium. In the past two decades the sexual cycle of a number of aspergilli could be induced when a range of media was tested, showing the requirement for very specific conditions [49]. For instance, *A. fumigatus* could be crossed on sealed oatmeal agar plates at 30 °C in the dark [24] while *A. parasiticus* [22], *Aspergillus nomius* [25] and *Aspergillus terreus* [23] were shown to form ascospores after several weeks on mixed cereal agar medium. Houbraken and Dyer gave an exhaustive overview of the different methods that can be used to induce a sexual cycle in filamentous fungi, with a particular focus on the use of different cultivation media and inoculation techniques [32]. Although a sexual cycle in *A. niger* has not been observed so far, the formation of sclerotia could be recently efficiently induced in some *A. niger* strains grown on organic substrates such as raisins or other plant parts [50].

## Three areas ripe for development

### Correlation between sexual reproduction and pathogenicity

The implications of sexual reproduction on the pathogenicity of fungal species has only been recently studied. Even if the available data is still limited, there are already indications that the ability of certain fungal species, including certain aspergilli, to undergo sexual development can have important consequences for pathogenesis [51]. Among the species belonging to the genus *Aspergillus*, most studies were performed on *A. fumigatus*, which is the most common causative agent of aspergillosis in immunocompromised patients [52]. Alvarez-Perez and colleagues suggested a possible correlation between the mating-type and the virulence of *A. fumigatus* strains, with an association between the MAT1-1 gene and isolates of invasive or clinical origin [53]. However, a subsequent study in which isogenic strains were used did not confirm the role of the mating-type in pathogenicity [54]. Therefore, further investigations are required to fully understand the role of the mating-type in pathogenic strains. Moreover, recombination derived from meiosis, by generating novel genotypes, might play a role in the increased pathogenicity and azole resistance of certain *A. fumigatus* isolates [18, 55]. Increased pathogenicity might also be related to the higher resistance of the ascospores, compared to conidia, to stress conditions [51], such as high temperatures [56]. Other *Aspergillus* species for which a sexual cycle is known, such as *A. flavus* and *A. parasiticus*, can be human or plant pathogens, can cause food spoilage and produce aflatoxins. Although a sexual cycle in *A. niger* has not been described yet, some strains were shown to be the causative agents of allergic reactions and infectious diseases, including aspergillosis, in immunocompromised patients [18, 57]. Therefore, further research focusing on the correlation between pathogenesis and (potential) sexual cycle in the aspergilli is required, due to the relevant medical implications that it would have to track pathogenesis and provide suitable treatments.

### Effect of climate change on the reproductive mode of the aspergilli

The influence of environmental conditions, including temperature, pH and humidity, on fungal growth and development is well known and characterized in the laboratory. The climate change, ongoing since the mid-twentieth century, has effects on the living organisms in their natural environment. However, studies on the effect of climate change on fungi are still limited and mostly focusing on basidiomycetes, because of the facility with which spore-bearing structures (mushrooms) can be monitored [58]. Changes in temperature and moisture have consequences on the reproductive season and on the timing of reproduction [59, 60]. Studies in the aspergilli are still very limited and they were mostly conducted on the aflatoxin producer *A. flavus*, focusing on the effects of increasing temperatures on aflatoxin production [61]. A study simulating the current and expected future increase in temperature was performed to investigate the response of different fungal species, including *A. niger* [62]. Higher growth rate and lower spore production can be expected in future climates [62]. Changes in climate not only affect mycelial growth, sporulation and toxin production but were also shown to cause a shift of wild species towards the poles [63]. Besides changing the current ecosystems, modifications in fungal growth and development raise concerns about food and health safety. For instance, altered levels of allergenic spores in the air might have an effect on the risk of patient sensitization [62] and colonization of new environments by plant and crop pathogens causes consequences in terms of global food security [63]. Global warming might bring new fungal infections as pathogenic species increase their geographic range and adapted thermotolerant strains are selected from ancestors with significant pathogenic potential but currently not pathogenic because suppressed by mammalian body heat [64]. The consequences can have a major impact on the ratio of mammals to reptiles as described by the FIMS hypothesis “fungal infection-mammalian selection” [65]. More studies are therefore required to analyze the relationship between climate change and modifications in the reproduction mode of fungi and its important implications.

### Implications of sexual reproduction in speciation studies

The study of the origin of species is a fundamental problem in biology that has puzzled scientists for decades and continues to be a topic of debate. In the past years, more than 20 different concepts of species have been proposed [66]. One of these is the biological species concept, which regards a species as a group of organisms where two individuals of opposite sexes can interbreed and produce fertile offspring [19]. In fungi, including in the genus *Aspergillus*, genetic and reproductive isolation seems to precede morphological differentiation [67]. In this regard, the study of speciation is not only a human-made concept but it assumes an important biological meaning, for which investigations of the reproduction modes and of the reproductive barriers present between organisms in nature are required. Recent discoveries of sexual cycle and characterization of the mating-type loci in members of the *Aspergillus* genus could provide new tools for studying the evolution of these species and the occurrence and the significance of cryptic species in nature. In the ascomycetes, the sequences flanking the MAT loci are highly conserved [29, 30, 68]. Therefore, the rate of conservation and evolution of the MAT loci and their flanking sequences provides a useful tool for speciation studies, allowing the inference of phylogenetic relationships between species of medical or industrial importance, in cases where commonly used barcode sequences alone are not enough [14, 69].

## Conclusions

The recent rapid development of genomic and genetic engineering technologies coupled with the identification of suitable conditions has led to the identification of sexual development in an increasing number of fungi previously considered asexual. Despite these advances, the sexual cycle of certain fungal species has not been discovered yet, although evidence of their sexual potential is accumulating. Examples include *A. niger* and *A. oryzae*, economically important species, for which the discovery of a sexual cycle would be of utmost relevance. Studies focusing on the natural ecology of these fungi, including their environmental conditions and their interaction partners in nature, are required to investigate the mode of reproduction in the members of the aspergilli and its significance for pathogenicity, environment and speciation.

## Supplementary information

**Additional file 1:** Poster: Fungal reproduction – asexual and sexual cycle in members of the genus *Aspergillus*.

## Data Availability

Not applicable.

## References

[1] Nieuwenhuis BPS, James TY (2016). The frequency of sex in fungi. Philos Trans R Soc B Biol Sci.

[2] Billiard S, López-Villavicencio M, Hood ME, Giraud T (2012). Sex, outcrossing and mating types: unsolved questions in fungi and beyond. J Evol Biol.

[3] Lee SC, Ni M, Li W, Shertz C, Heitman J (2010). The evolution of sex: a perspective from the fungal kingdom. Microbiol Mol Biol Rev.

[4] Todd RB, Davis MA, Hynes MJ (2007). Genetic manipulation of *Aspergillus nidulans*: Meiotic progeny for genetic analysis and strain construction. Nat Protoc.

[5] de Visser JAGM, Elena SF (2007). The evolution of sex: empirical insights into the roles of epistasis and drift. Nat Rev Genet.

[6] Felsenstein J (1974). The evolutionary advantage of recombination. Genetics.

[7] Otto SP, Lenormand T (2002). Resolving the paradox of sex and recombination. Nat Rev Genet.

[8] Hurst LD, Peck JR (1996). Recent advances in understanding of the evolution and maintenance of sex. Trends Ecol Evol.

[9] Varga J, Szigeti G, Baranyi N, Kocsubé S, O’Gorman CM, Dyer PS (2014). *Aspergillus*: sex and recombination. Mycopathologia.

[10] Dyer PS, Kück U. Sex and the imperfect fungi. The Fungal Kingdom. 2017;193–214. 10.1128/microbiolspec.funk-0043-2017 PMC11687501 28597816

[11] Dyer PS, O’Gorman CM (2012). Sexual development and cryptic sexuality in fungi: insights from *Aspergillus* species. FEMS Microbiol Rev.

[12] Geiser DM (2009). Sexual structures in *Aspergillus*: morphology, importance and genomics. Med Mycol.

[13] Dyer PS, O’Gorman CM (2011). A fungal sexual revolution: *Aspergillus* and *Penicillium* show the way. Curr Opin Microbiol.

[14] Pöggeler S (2001). Mating-type genes for classical strain improvements of ascomycetes. Appl Microbiol Biotechnol.

[15] Kück U, Böhm J (2013). Mating type genes and cryptic sexuality as tools for genetically manipulating industrial molds. Appl Microbiol Biotechnol.

[16] Plan D, Van Den Eede G. The EU legislation on GMOs - An overview. 2010

[17] European Parliament Council of the European Union. Regulation (EC) No 1829/2003 of the European Parliament and of the Council of 22 September 2003 on genetically modified food and feed (Text with EEA relevance). 1829/2003 Official Journal of the European Union; 2003

[18] Paulussen C, Hallsworth JE, Álvarez-Pérez S, Nierman WC, Hamill PG, Blain D (2017). Ecology of aspergillosis: insights into the pathogenic potency of *Aspergillus fumigatus* and some other *Aspergillus* species. Microb Biotechnol.

[19] Dobzhansky TG (1934). Genetics and the origin of the species. Philos Sci.

[20] Fennell DI, Warcup JH (1959). The ascocarps of *Aspergillus alliaceus*. Mycologia.

[21] Horn BW, Moore GG, Carbone I (2009). Sexual reproduction in *Aspergillus flavus*. Mycologia.

[22] Horn BW, Ramirez-Prado JH, Carbone I (2009). The sexual state of *Aspergillus parasiticus*. Mycologia.

[23] Arabatzis M (2013). Sexual reproduction in the opportunistic human pathogen Aspergillus terreus. Mycologia.

[24] O’Gorman CM, Fuller HT, Dyer PS (2009). Discovery of a sexual cycle in the opportunistic fungal pathogen *Aspergillus fumigatus*. Nature.

[25] Horn BW, Moore GG, Carbone I (2011). Sexual reproduction in aflatoxin-producing *Aspergillus nomius*. Mycologia.

[26] Horn BW, Olarte RA, Peterson SW, Carbone I (2013). Sexual reproduction in *Aspergillus tubingensis* from section *Nigri*. Mycologia.

[27] Sharma KK (2016). Fungal genome sequencing: basic biology to biotechnology. Crit Rev Biotechnol.

[28] Pel HJ, de Winde JH, Archer DB, Dyer PS, Hofmann G, Schaap PJ (2007). Genome sequencing and analysis of the versatile cell factory *Aspergillus niger* CBS 513.88. Nat Biotechnol.

[29] Galagan JE, Hynes M, Pain A, Machida M, Purcell S, Peñalva MÁ (2005). Sequencing of *Aspergillus nidulans* and comparative analysis with *A. fumigatus* and *A. oryzae*. Nature.

[30] Dyer PS. Sexual reproduction and significance of MAT in the Aspergilli. In: Heitman J, Kronstad J, Taylor J CL (ed), editor. Sex Fungi. ASM Press. American Society of Microbiology; 2007. p. 123–42. 10.1128/9781555815837.ch7.

[31] de Vries RP, Riley R, Wiebenga A, Aguilar-Osorio G, Amillis S, Uchima CA (2017). Comparative genomics reveals high biological diversity and specific adaptations in the industrially and medically important fungal genus *Aspergillus*. Genome Biol.

[32] Houbraken J, Dyer PS. Induction of the sexual cycle in filamentous ascomycetes. In: van den Berg MA, Maruthachalam K, editors. Genet Transform Syst Fungi, Vol 2. Cham: Springer International Publishing; 2015. p. 23–46. 10.1007/978-3-319-10503-1_2.

[33] Zhang X, Tan Y, Yu F, Liu Z, Ge Y. Comparative transcriptome sequence analysis of sporulation-related genes of *Aspergillus cristatus* in response to low and high osmolarity. Curr Microbiol. Springer US; 2017;74:806–14. 10.1007/s00284-017-1250-x 28417188

[34] Andersen MR, Salazar MP, Schaap PJ, van de Vondervoort PJI, Culley D, Thykaer J (2011). Comparative genomics of citric-acid-producing *Aspergillus niger* ATCC 1015 versus enzyme-producing CBS 513.88. Genome Res.

[35] Engler C, Gruetzner R, Kandzia R, Marillonnet S (2009). Golden Gate Shuffling: A one-pot DNA shuffling method based on type IIs restriction enzymes. PLoS ONE.

[36] Engler C, Kandzia R, Marillonnet S (2008). A One pot, one step, precision cloning method with high throughput capability. PLoS ONE.

[37] Weber E, Engler C, Gruetzner R, Werner S, Marillonnet S (2011). A modular cloning system for standardized assembly of multigene constructs. PLoS ONE.

[38] Sarkari P, Marx H, Blumhoff ML, Mattanovich D, Sauer M, Steiger MG (2017). An efficient tool for metabolic pathway construction and gene integration for *Aspergillus niger*. Bioresour Technol Elsevier Ltd.

[39] Nødvig CS, Nielsen JB, Kogle ME, Mortensen UH (2015). A CRISPR-Cas9 system for genetic engineering of filamentous fungi. PLoS ONE.

[40] Meyer V, Wanka F, van Gent J, Arentshorst M, van den Hondel CAMJJ, Ram AFJ. Fungal gene expression on demand: an inducible, tunable, and metabolism-independent expression system for *Aspergillus niger*. Appl Environ Microbiol. 2011;77:2975–83. 10.1128/aem.02740-10. 10.1128/AEM.02740-10 PMC3126388 21378046

[41] Pyrzak W, Miller KY, Miller BL (2008). Mating type protein Mat1-2 from asexual *Aspergillus fumigatus* drives sexual reproduction in fertile *Aspergillus nidulans*. Eukaryot Cell.

[42] Große V, Krappmann S (2008). The sexual pathogen *Aspergillus fumigatus* expresses functional determinants of *Aspergillus nidulans* sexual development. Eukaryot Cell.

[43] Wada R, Maruyama JI, Yamaguchi H, Yamamoto N, Wagu Y, Paoletti M (2012). Presence and functionality of mating type genes in the supposedly asexual filamentous fungus *Aspergillus oryzae*. Appl Environ Microbiol.

[44] Yu Y, Blachowicz A, Will C, Szewczyk E, Glenn S, Gensberger-Reigl S (2018). Mating-type factor-specific regulation of the fumagillin/pseurotin secondary metabolite supercluster in *Aspergillus fumigatus*. Mol Microbiol.

[45] Jørgensen TR, Burggraaf A-M, Arentshorst M, Schutze T, Lamers G, Niu J (2020). Identification of SclB, a Zn(II)2Cys6 transcription factor involved in sclerotium formation in *Aspergillus niger*. Fungal Genet Biol.

[46] Rai JN, Tewari JP, Sinha AK (1967). Effect of environmental conditions on sclerotia and cleistothecia production in *Aspergillus*. Mycopathol Mycol Appl.

[47] Agnihotri VP (1968). Some nutritional and environmental factors affecting growth and production of sclerotia by a strain of *Aspergillus niger*. Can J Microbiol.

[48] Han KH, Lee DB, Kim JH, Kim MS, Han KY, Kim WS (2003). Environmental factors affecting development of *Aspergillus nidulans*. J Microbiol.

[49] Kwon-Chung KJ, Sugui JA (2009). Sexual reproduction in *Aspergillus* species of medical or economical importance: why so fastidious?. Trends Microbiol.

[50] Frisvad JC, Petersen LM, Lyhne EK, Larsen TO (2014). Formation of sclerotia and production of indoloterpenes by *Aspergillus niger* and other species in section *Nigri*. PLoS ONE.

[51] Ene IV, Bennett RJ (2014). The cryptic sexual strategies of human fungal pathogens. Nat Rev Microbiol Nature Publishing Group.

[52] Latgé JP (1999). *Aspergillus fumigatus* and aspergillosis. Clin Microbiol Rev.

[53] Alvarez-Perez S, Blanco JL, Alba P, Garcia ME (2010). Mating type and invasiveness are significantly associated in *Aspergillus fumigatus*. Med Mycol.

[54] Losada L, Sugui JA, Eckhaus MA, Chang YC, Mounaud S, Figat A (2015). Genetic analysis using an isogenic mating pair of *Aspergillus fumigatus* identifies azole resistance genes and lack of MAT locus’s role in virulence. PLOS Pathog.

[55] Camps SMT, Rijs AJMM, Klaassen CHW, Meis JF, O’Gorman CM, Dyer PS (2012). Molecular epidemiology of *Aspergillus fumigatus* isolates harboring the TR34/L98H azole resistance mechanism. J Clin Microbiol.

[56] Snyder AB, Biango-Daniels MN, Hodge KT, Worobo RW (2019). Nature abhors a vacuum: highly diverse mechanisms enable spoilage fungi to disperse, survive, and propagate in commercially processed and preserved foods. Compr Rev Food Sci Food Saf.

[57] Schuster E, Dunn-Coleman N, Frisvad J, van Dijck P (2002). On the safety of *Aspergillus niger* - a review. Appl Microbiol Biotechnol.

[58] Bidartondo MI, Ellis C, Kauserud H, Kennedy PG, Lilleskov EA, Suz LM, et al. Climate change: Fungal responses and effects. State of the World’s Fungi. 2018. p. 62–9.

[59] Boddy L, Büntgen U, Egli S, Gange AC, Heegaard E, Kirk PM (2014). Climate variation effects on fungal fruiting. Fungal Ecol.

[60] Sato H, Morimoto S, Hattori T (2012). A thirty-year survey reveals that ecosystem function of fungi predicts phenology of mushroom fruiting. PLoS ONE.

[61] Medina A, Rodriguez A, Magan N (2014). Effect of climate change on *Aspergillus flavus* and aflatoxin B1 production. Front Microbiol.

[62] Damialis A, Mohammad AB, Halley JM, Gange AC (2015). Fungi in a changing world: growth rates will be elevated, but spore production may decrease in future climates. Int J Biometeorol.

[63] Bebber DP, Ramotowski MAT, Gurr SJ (2013). Crop pests and pathogens move polewards in a warming world. Nat Clim Chang.

[64] Garcia-Solache MA, Casadevall A (2010). Global warming will bring new fungal diseases for mammals. MBio.

[65] Casadevall A, Damman C (2020). Updating the fungal infection-mammalian selection hypothesis at the end of the Cretaceous Period. PLOS Pathog.

[66] Hey J (2001). The mind of the species problem. Trends Ecol Evol.

[67] Taylor JW, Turner E, Townsend JP, Dettman JR, Jacobson D (2006). Eukaryotic microbes, species recognition and the geographic limits of species: examples from the kingdom Fungi. Philos Trans R Soc B Biol Sci.

[68] Debuchy R, Turgeon BG. Mating-type structure, evolution, and function in Euascomycetes. Growth, Differ Sex. Growth, Di. Berlin/Heidelberg: Springer-Verlag; 2006. p. 293–323. 10.1007/3-540-28135-5_15.

[69] Pál K, Van Diepeningen AD, Varga J, Hoekstra RF, Dyer PS, Debets AJM (2007). Sexual and vegetative compatibility genes in the aspergilli. Stud Mycol.

