# Supplementary material for: The fungal sexual revolution continues: discovery of sexual development in members of the genus Aspergillus and its consequences
Source: Fungal Biol Biotechnol. 2020 Dec 24;7:17. doi: 10.1186/s40694-020-00107-y (PMC7761153; doi:10.1186/s40694-020-00107-y)
Supplement: Supplementary file 1 — Additional file 1: Poster: Fungal reproduction – asexual and sexual cycle in members of the genus Aspergillus. [file 40694_2020_107_MOESM1_ESM.pptx]

## Slide 1
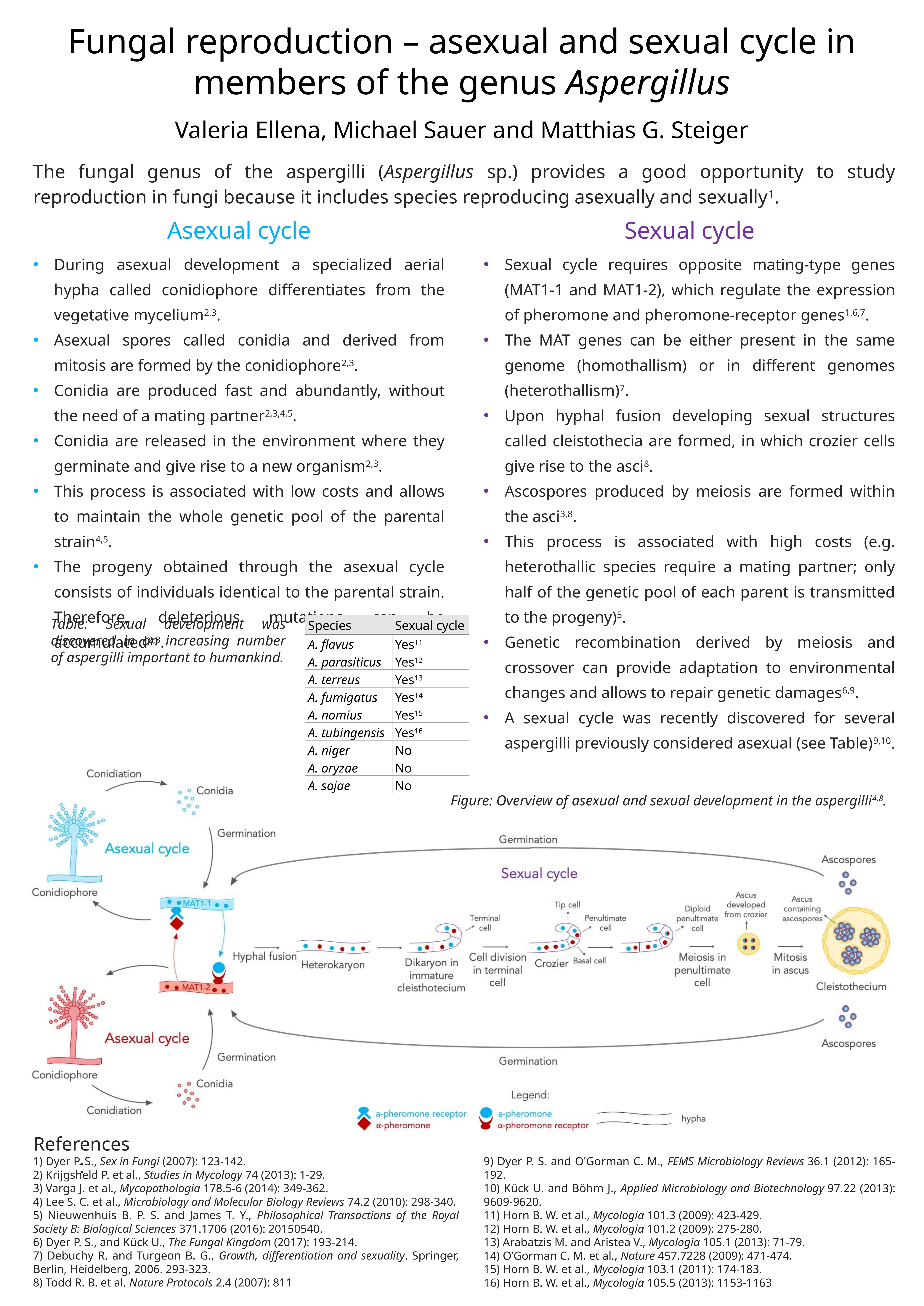

Fungal reproduction – asexual and sexual cycle in members of the genus Aspergillus
Valeria Ellena, Michael Sauer and Matthias G. Steiger
The fungal genus of the aspergilli (Aspergillus sp.) provides a good opportunity to study reproduction in fungi because it includes species reproducing asexually and sexually1.
Asexual cycle
During asexual development a specialized aerial hypha called conidiophore differentiates from the vegetative mycelium2,3.
Asexual spores called conidia and derived from mitosis are formed by the conidiophore2,3.
Conidia are produced fast and abundantly, without the need of a mating partner2,3,4,5.
Conidia are released in the environment where they germinate and give rise to a new organism2,3.
This process is associated with low costs and allows to maintain the whole genetic pool of the parental strain4,5.
The progeny obtained through the asexual cycle consists of individuals identical to the parental strain. Therefore, deleterious mutations can be accumulated2,3.
Sexual cycle
Sexual cycle requires opposite mating-type genes (MAT1-1 and MAT1-2), which regulate the expression of pheromone and pheromone-receptor genes1,6,7.
The MAT genes can be either present in the same genome (homothallism) or in different genomes (heterothallism)7.
Upon hyphal fusion developing sexual structures called cleistothecia are formed, in which crozier cells give rise to the asci8.
Ascospores produced by meiosis are formed within the asci3,8.
This process is associated with high costs (e.g. heterothallic species require a mating partner; only half of the genetic pool of each parent is transmitted to the progeny)5.
Genetic recombination derived by meiosis and crossover can provide adaptation to environmental changes and allows to repair genetic damages6,9.
A sexual cycle was recently discovered for several aspergilli previously considered asexual (see Table)9,10.
Table: Sexual development was discovered in an increasing number of aspergilli important to humankind.
| Species | Sexual cycle |
| --- | --- |
| A. flavus | Yes11 |
| A. parasiticus | Yes12 |
| A. terreus | Yes13 |
| A. fumigatus | Yes14 |
| A. nomius | Yes15 |
| A. tubingensis | Yes16 |
| A. niger | No |
| A. oryzae | No |
| A. sojae | No |
Figure: Overview of asexual and sexual development in the aspergilli4,8.
References:
1) Dyer P. S., Sex in Fungi (2007): 123-142.
2) Krijgsheld P. et al., Studies in Mycology 74 (2013): 1-29.
3) Varga J. et al., Mycopathologia 178.5-6 (2014): 349-362.
4) Lee S. C. et al., Microbiology and Molecular Biology Reviews 74.2 (2010): 298-340.
5) Nieuwenhuis B. P. S. and James T. Y., Philosophical Transactions of the Royal Society B: Biological Sciences 371.1706 (2016): 20150540.
6) Dyer P. S., and Kück U., The Fungal Kingdom (2017): 193-214.
7) Debuchy R. and Turgeon B. G., Growth, differentiation and sexuality. Springer, Berlin, Heidelberg, 2006. 293-323.
8) Todd R. B. et al. Nature Protocols 2.4 (2007): 811
9) Dyer P. S. and O'Gorman C. M., FEMS Microbiology Reviews 36.1 (2012): 165-192.
10) Kück U. and Böhm J., Applied Microbiology and Biotechnology 97.22 (2013): 9609-9620.
11) Horn B. W. et al., Mycologia 101.3 (2009): 423-429.
12) Horn B. W. et al., Mycologia 101.2 (2009): 275-280.
13) Arabatzis M. and Aristea V., Mycologia 105.1 (2013): 71-79.
14) O’Gorman C. M. et al., Nature 457.7228 (2009): 471-474.
15) Horn B. W. et al., Mycologia 103.1 (2011): 174-183.
16) Horn B. W. et al., Mycologia 105.5 (2013): 1153-1163.
